# Do informal caregivers for elderly in the community use support measures? A qualitative study in five European countries

**DOI:** 10.1186/s12913-016-1487-2

**Published:** 2016-07-16

**Authors:** Evi Willemse, Sibyl Anthierens, Maria Isabel Farfan-Portet, Olivier Schmitz, Jean Macq, Hilde Bastiaens, Tinne Dilles, Roy Remmen

**Affiliations:** University of Antwerp, Antwerp, Belgium; Thomas More University College, Turnhout, Belgium; Université Catholique De Louvain, Louvain, Belgium; Belgian Health Care Knowledge Centre (KCE), Bruxelles, Belgium

**Keywords:** (Family) caregivers, Continuity of care, Long term care, Primary care, Support

## Abstract

**Background:**

Informal caregivers are essential figures for maintaining frail elderly at home. Providing informal care can affect the informal caregivers’ physical and psychological health and labour market participation capabilities. They need support to prevent caregiver burden. A variety of existing support measures can help the caregiver care for the elderly at home, but with some limitations. The objective of this review was to explore the experiences of informal caregivers caring for elderly in the community with the use of supportive policy measures in Belgium and compare these to the experiences in other European countries.

**Methods:**

An empirical qualitative case study research was conducted in five European countries (Belgium, The Netherlands, Luxembourg, France and Germany). Semi-structured interviews were conducted with informal caregivers and their dependent elderly. Interview data from the different cases were analysed. In particular data from Belgium was compared to data from the cases abroad.

**Results:**

Formal services (e.g. home care) were reported to have the largest impact on allowing the caregiver to care for the dependent elderly at home. One of the key issues in Belgium is the lack of timely access to reliable information about formal and informal services in order to proactively support the informal caregiver. Compared to the other countries, informal caregivers in Belgium expressed more difficulties in accessing support measures and navigating through the health system. In the other countries information seemed to be given more timely when home care was provided via care packages.

**Conclusion:**

To support the informal caregiver, who is the key person to support the frail elderly, fragmentation of information regarding supportive policy measures is an important issue of concern.

**Electronic supplementary material:**

The online version of this article (doi:10.1186/s12913-016-1487-2) contains supplementary material, which is available to authorized users.

## Background

The European population is aging rapidly, and the number of very old people in particular will increase in the coming decades. Aging is accompanied by a decrease in health and an increase in the number of chronic diseases. It is expected that the ageing population will increase the need and consumption of long-term care in Europe over time [1, 2].

According to the Organisation for Economic Cooperation and Development (OECD), long-term care is “a range of services required by persons with a reduced degree of functional capacity, physical or cognitive, and who are consequently dependent for an extended period of time on help with basic activities of daily living (ADL)”. Long term care includes both formal and informal care [3]. Formal care refers to provisions to dependent people by health and social care professionals within regulated employment relationships. Informal care refers to the care or support given on a voluntary basis to a dependent elderly by a family member, friend or acquaintance [3].

In Belgium and in other OECD countries, there is a growing awareness of the importance of informal care for the organisation of the present and future health care system [4]. The need for support and the need for a coordinated and integrated approach for elderly care is highlighted in several studies in Belgium [4, 5]. The care transition process, with an increasing attention for informal home care, offers a critical opportunity to treat informal caregivers as important care partners [5]. The family is described as the biggest source of help for the elderly and for the sustainability of our chronic health care system [4].

However, providing informal care can affect the informal caregivers’ physical and psychological health and labour market participation capabilities [6]. In order to counter the possible negative impact of providing informal care on these aspects, policies have been implemented in different countries to provide financial support, improve the balance between working and caring, and enhance the informal caregiver’s wellbeing [3].

In recent years, several studies have provided detailed descriptions of available policies in different countries [3, 7]. The studies point out that the way in which support is actually provided does not correspond with one single pathway and that the effectiveness of the measures is not easily assessed [7]. The latter may be partially related to the fact that measures to provide informal caregivers with support are scattered in different sectors of the social security system. Due to the diversity in caregivers’ needs, the uncertainty on the effectiveness of measures and the complexity of social security systems it is difficult to create a comprehensive policy framework responding to the needs of all informal caregivers. Yet, there is some evidence that a comprehensive set of measures and integrated support packages tailored to the individual needs are required to support caregivers of frail elderly in order to prevent caregiver burden [8, 9].

This study builds an innovative approach to the evaluation of support measures for informal caregivers using a single and comprehensive framework to assess existing policy measures in five countries, i.e. Belgium, France, Germany, Luxembourg and the Netherlands [7]. In particular data from Belgium is compared to data from the cases abroad.

## Methods

### Design of the study and setting

**Fig. 1:**
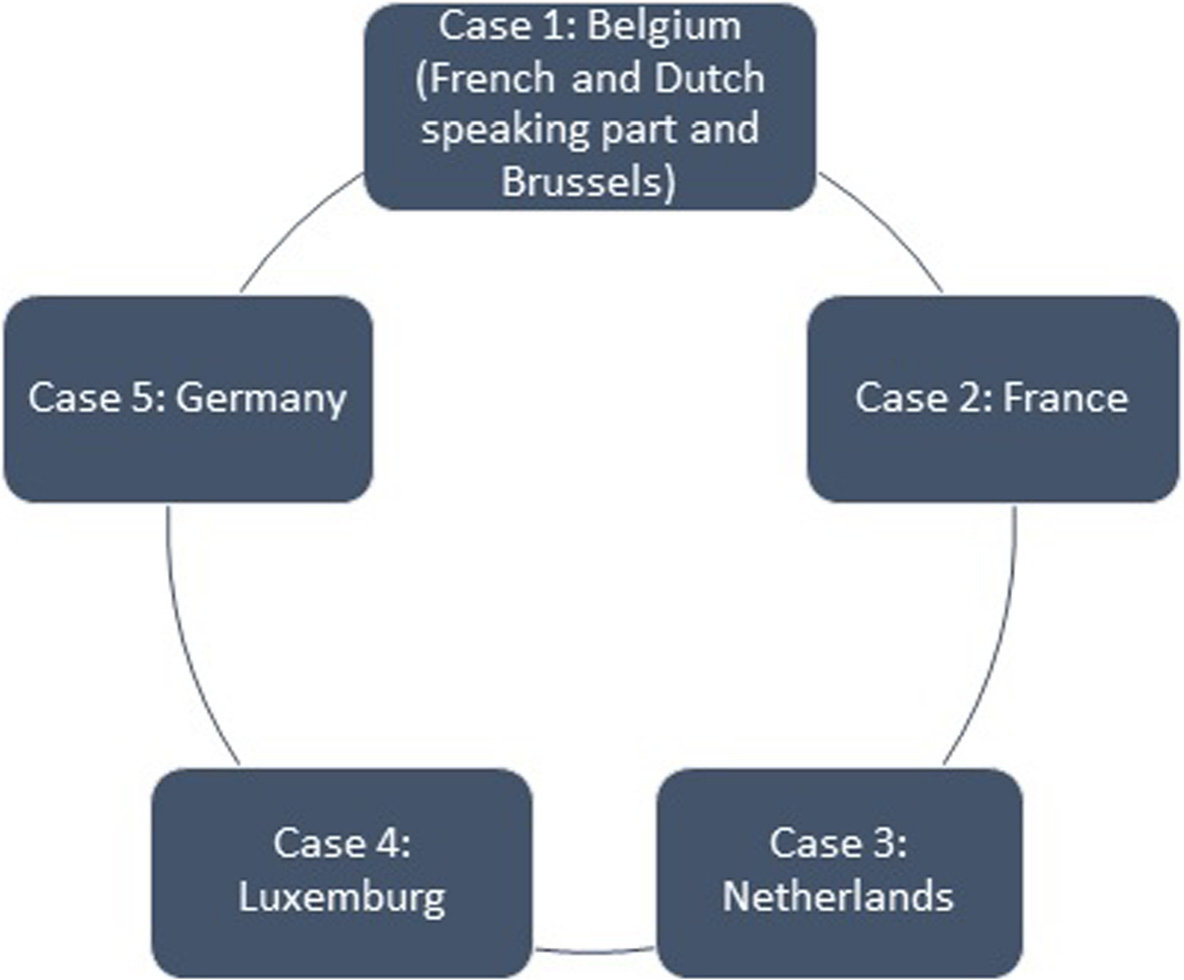
Embedded multiple case study design

A multiple case study design was used to explore the different experiences of care in relation to their knowledge and use of policy measures [10]. Case study research is a qualitative approach in which the investigator explores multiple bounded systems (cases) through detailed, in-depth data collection, and reports a case description and case-based themes [10]. Multiple case studies allow for comparison, particularly in diverse settings. In this regard, we described and compared the experiences and perceptions of informal caregivers in five different countries (Fig. 1).

Each case contains subunits of analysis (the participants) consisting of the dependent elderly and his/her main informal caregiver. For each subunit, we studied the informal caregivers’ and the dependent elderly’s experiences and perceptions of support services provided to them by the different competent authorities. The differences within the cases, divided in a seven topic framework (see Additional file 1) were constructed to assess the different support measures identified in the different countries. A detailed description of the policy measures can be found in Anthierens et al. [7]. All cases were analysed, with a special focus on Belgium with the aim of contributing towards a new policy for informal caregivers.

### Study participants

Recruitment guidelines for informal caregivers and their dependent elderly (subunits) were developed (Table 1). Heterogeneity in family relationships, trajectories in the labour-market and formal service use was pursued. Subunits had to represent common cases, defined as frequently seen by general practitioners. Persons who were receiving palliative care, hospitalised in the past four weeks or having an acute disease, were excluded. Five informal caregivers per case and at least three dependent elderly had to be interviewed. When it was possible, elderly were interviewed separately (after the caregiver), to avoid mutual influence of each other during the interviews. The interviews lasted approximately one hour. Recruitment, by phone contact or face to face meeting, was carried out by local General Practitioners (GP), nursing services, elderly homes or social services. All the respondents signed informed consent and agreed upon audio taping, subsequent analysis and publication of the data.

**Table 1:** Sample frame of subunits

1. A necessary condition for inclusion of ICG and DEP is having the ability to carry out the interviews separately with the DEP and the ICG. So, it is important that the GP or the person who contacts the informal caregiver and his/her dependent elderly asks whether this is possible before the interview takes place.
2. Ideally, we aim to interview subunits but in some situations, it will not be possible to interview the dependent elderly. For each case study, we have to interview at least 3 subunits.
3. When it is possible to interview the subunit, it is important to have the first interview with the caregiver, in order not to burden the dependent elderly with too many questions (that we have already covered with the caregiver).
4. It is important for the GP who recruits the subunits that he/she has ‘common situations’ in mind (cases that occur often in the practice, not the exceptions) (ideally we recruit people with a variety of medical conditions but we need at least 2 people with dementia). The caregiver has taken up his/her role for at least 1 year. Common cases should include subunits receiving a lot of formal services as well as little or no services at all according to the GP. This can apply to people living in the community or in an institution (level of services refers to the situation before institutionalization).
5. In addition to this, the dependent elderly needs to be 70 years or older and not being : o in palliative care. o hospitalised in the past four weeks o having an acute disease
6. We keep a mix between spouse, child caregivers (the exact number will be defined by the sampling)
7. Two dependent elderly institutionalized but less than four months (at least 1 people with dementia), Three in the community (at least 1 people with dementia).
8. Select at least two caregivers who are in paid employment.
9. At least 1 man as caregiver

### Data collection

An interview guide for semi-structured interviews was developed in English. A first version of the guide for the elderly living in the community was prepared by three researchers and separate semi-structured guides were constructed for the institutionalised elderly. The first version of the guide was compared with three recent surveys in order to ensure that all relevant topics were included (e.g. basic questionnaire, definition of care tasks) [11–13]. The new version was then reviewed by the research team. Afterwards, questions were translated in English and then translated again into the languages of the participating countries.

The interview guide consisted of open ended questions (Appendix Table 3). Questions and interview topics were based on an analysis of existing policies (formal and informal) available to caregivers in their respective country [7].

Five experienced researchers, one for each country, conducted the interviews. For each case, the analysis was done using the framework and was then discussed within the team, each researcher was a native speaker of the language used during the interview. In order to increase uniformity in all cases, peer debriefing occurred before starting the interviews. The first interview from one subunit in each case was discussed by all members of the research team. The team discussed data between the members of the research team during four meetings.

### Data analysis

In order to have a single analysis framework, a grid for the analysis of the interviews was developed based on two pilot interviews in Belgium. The grid was then sent to the five researchers conducting the interviews. The first interview in each subunit (caregiver and dependent elderly) was encoded to appraise the analysis grid validity. The analysis grid was divided into four parts: (1) description of the family, professional and living situation of the informal caregiver and the dependent elderly, (2) informal caregiver role, (3) formal services for the dependent elderly, (4) support measures for the informal caregiver. The analysis grid could evolve during the collecting of data and any changes were communicated between the researchers. A reporting scheme that included some representative quotes in their respective language was used to report the findings. After analysing the separate cases, thematic analysis was performed across cases to define domains.

## Results

The interview data was divided into four parts (1) description of the family, professional and living situation of the informal caregiver and the dependent elderly, (2) the informal caregiver role, (3) formal services for the dependent elderly, and (4) support measures for the informal caregivers.

### Part 1: Description of the family, professional and living situation of the informal caregiver and the dependent elderly

A total of 35 informal caregivers and their dependent elderly were interviewed. For each case at least five informal caregivers and three dependent elderly were approached. The additional information collected from the interviews with the dependent elderly, that were held separately (after the interview with the caregiver) was little (because of the condition of the dependent elderly). The interviews with the dependent elderly yielded no new information for any of the cases. Characteristics of the participants are reported in Table 2.

**Table 2:** Demographics of the subunits/participants

Informal caregivers demographics per case (*n* = 35)	Belgium (*n* = 15)	France (*n* = 5)	Netherlands (*n* = 5)	Germany (*n* = 5)	Luxembourg (*n* = 5)
Female	14	4	3	4	4
Mean age (years)	56.8	63	55.4	69	74
Married	14	4	2	5	5
Widowed	1				
Single/Divorced	2	1	3		
Family relationship^a^	16	4	5	5	5
Living/has lived with DEP	9	2	2	4	3
In the labour market	10	2	5	1	0
Dependent elderly demographics per case (*n* = 35)	Belgium (*n* = 15)	France (*n* = 5)	Netherlands (*n* = 5)	Germany (*n* = 5)	Luxembourg (*n* = 5)
Female	11	5	4	3	2
Mean age (years)	81	88.4	71.6	80.6	85.2
Married	5	1		3	4
Widowed	12	4	4	2	1
Family relationship^b^	17	4	5	5	5
Living at home	13	3	3	3	4
Dementia/Parkinson	8	2	2	2	1

^a^husband, spouse, cousin, daughter (-in law), son

^b^mother (-in law), father, cousin, husband, wife

### Dependent elderly demographics

Dependent elderly were over 70 (mean 85 years). The dependent person was slightly younger (66 and 69 years old) in two subunits in the Belgian case. The majority of the elderly were female, with the exception of the Luxembourg case (three of the five were male). Most caregivers were family members. Care was usually provided by the spouse or by a child (sometimes child-in-law). The informal caregivers shared the house with the partner or wife/husband in subunits where the elderly was married and not institutionalized. In the Netherlands, Germany, Belgium and France, a mix of co-residence status with child (or in-law) caregivers was present.

### Caregiver demographics

The average age of the caregivers (spouse/partner relationship with the dependent older person) was 61 years. The mean age for non-spouse caregivers was 53 years. Regarding active job occupation the sample was heterogeneous, except for Luxembourg where the sample was older and all caregivers were retired. In the Netherlands, Germany, Wallonia and France, a mix of co-residence status with child(-in-law) caregivers was present. In Belgium (French speaking part) all daughters providing care lived-in with the dependent older person.

### Part 2: The informal caregiver role

Caregivers played an essential role in arranging and managing the continuity of care of the dependent elderly. In all cases, caregivers to some extent provide personal care or support at home and ensure the continuity of care through coordination of formal care. From our data, it seemed that the caregivers in all cases preferred to do as much as possible without professional help. The caregiver was often the key person to provide care and coordination for the dependent elderly.

In all cases, the caregivers showed a high degree of resilience towards being a care provider. The caregivers often mentioned the reciprocity principle (i.e., the mutual obligation of the members from the same family to look after each other at different moments in life). They take on this role, especially when the caregiver is a child of the dependent older person.

*Q1: “I know that there is new arrangement for informal caregivers. But I haven’t looked any further into it. It’s not of much importance. We did look for support with devices, that sort of thing you follow up.” (caregiver the Netherlands).*

Furthermore, in all cases, the caregiver and even more so the dependent elderly preferred being cared for at home by someone they knew rather than being institutionalised. This allowed the dependent elderly a larger level of autonomy, to maintain the home feeling, and to have a say in the care they receive.

*Q2: “Providing informal care and making decisions are done in good harmony with her (dependent elderly). She (elderly person) was able to indicate to us clearly what she expected from us and what she didn’t. For example, bathing in the shower or bath was left to a professional caregiver. The choices were made together with her.” (caregiver Belgium).*

Yet, sometimes coordination of care comes at a high cost and is an unbalanced equilibrium. If caregivers can no longer fulfil this role, other family members, friends, and neighbours are called to ensure the continuity of care.

*Q3: “So I told my brothers, you have to go and check on mother one or two extra times, as we’re going on holiday. And that’s no problem.” (caregiver Netherlands).*

Unfortunately, not all caregivers have support from their surroundings and this can have an impact on the level of emotional burden*.*

*Q4: “This year I went on holiday to the seaside with my child. I was making myself comfortable when I got a call from the social worker to tell me she could not provide care during the three days a week while I was at the sea … She had a problem with her staff … I thought I was going to die, and then, no humanity, no one apologizes …" (caregiver Belgium).*

*Q5: “Mom has always been a woman who was very independent. Every day she could go out whenever she wanted to. Now everything has changed, she is the caregiver, and has all the weight on her shoulders. She does not know when she can go out. She needs to look after him all the time, she cannot lock him (her husband) up in the house and go out …" (caregiver Belgium).*

### Part 3: Formal services for the dependent elderly

Most caregivers are informed about the formal services available to the dependent elderly. Information (e.g. home care and nursing care) is provided by the general practioner or the social assistant at discharge from hospital.

In the Netherlands, Luxembourg, Germany and France, care for the dependent elderly, and information about this care, was offered in a care package by health insurance or by the municipality. In contrast to Belgium where services for the dependent elderly and also information on these services were divided between several sectors of the social security system. Finding information seemed to be more difficult in Belgium.

In Germany, all the caregivers were aware of existing support measures for the dependent elderly and for the caregiver and used them extensively. In Luxembourg, accessibility to the available measures had been made transparent through formal media, where information was provided by health insurances. In the Netherlands and France, the dependent elderly and the caregiver received information through a central contact person from the health insurance. The caregivers reported that the whole process of caring for the elderly had been mediated by health and social care professionals (e.g. social worker, nurse, general practitioner, et cetera*).*

*Q6: “It all went extremely fast, there was a man from Assurance Dependence that came to assess the situation and the degree of burden, then the caregiver only had to sign. The next month the money was there.” (caregiver Luxembourg).*

### Part 4: Support measures for informal caregivers

For the majority of the caregivers and their dependent elderly person in Belgium, the formal services provided for the dependent elderly were the only source of support available. Informal caregivers expressed that access to formal care services for the dependent older person is hindered by how difficult it is to find one’s way into the system as well as by the cost of services. However, all of the dependent older people received some form of formal care within their home. The use of formal services seemed to be dependent on the financial situation of the caregiver and whether the caregiver was still in the labour force.

*Q7: "They live relatively well, they are not in need of for more help. But if you have to use more nursing assistance, I do not think is it possible … From the care insurance, he receives 343 euros, it helps to cover medical expenses. He already needs almost 300 euros for drugs per month. Also 300 euros per month for the day centre, and he only has a pension of 900 euros, so if he needs nursing help or if he wants to give a contribution to the informal caregivers, he has nothing to eat …" (caregiver Belgium).*

In the Belgian case, in all subunits, when caregivers are still active in the labour force, they are looking for more professional help themselves. They use measures targeting income, flexible work arrangements and leave policies. The interviewed informal caregivers mentioned that having a flexible work schedule was enough to cope with the care needs.

*Q8: “It's a bit like a fixed schedule, but you could be flexible with it. They saw to it at work that I was able to switch shifts with colleagues. They are very nice colleagues, they have taken my situation into account.” (caregiver Belgium).*

Flexible work arrangements and leave policies are used to support the caregiver in looking after the dependent elderly and to sustain their role and thus the continuity of care for the elderly.

Financial compensation services, e.g. caregiver allowance, were not always provided in the city/village of most of our respondents. The municipalities can choose whether they provide financial compensations for informal caregivers or not. If caregiver allowances were available, they were seldom requested because they were often deemed too small to cover the costs for the caregiver or because of the administrative requirements in order to receive the allowance. The allowance was nice to have but did not influence the decision to provide care or not.

Q9: “*I find it important to home-care. And you should not be paid. But I can understand if you do not work or do not have work because you home-care, then it's another matter.” (caregiver Netherlands)*

*Q10: “The amount is not the same everywhere. In one place they get 50 euros, 20 euros at the other. That's a big difference. And here they get nothing. I (DEP) asked, can’t they then have euros for a bunch of flowers? I think that we need to appreciate them and volunteering is not appreciated, it's because you’re happy to do it. You won’t get anything for doing it.” (caregiver Flanders)*

*Q11: “Well, an informal care allowance, two hundred euros per year, we just received that. We went out for dinner with the four of us with that money; my brother, his wife and us two. Because they also help our mother.” (caregiver Netherlands).*

Policy measures, such as respite care and psychosocial support, were seldom used or known.

Informal caregivers reported that when respite care alternatives were available, they were seldom adapted to the needs or preferences of the informal caregiver and/or the dependent older person. For other informal caregivers and dependent older people, it appeared unacceptable to use these types of services. The reasons given were a lack of trust and the strong relationship between the informal caregiver and the dependent older person.

Also, if respite care and psychosocial support were known to them, there was often a financial barrier (i.e. the amount of money paid for respite care was too high).

*Q12: “The amount that you get every month from the care allowance, you can use for groceries and then it’s gone, with that small amount you cannot cover all financial things. If you need to look for help everywhere and everything has to be paid for, then you need to have some savings.” (Caregiver Belgium)*

This in contrast to the subunits from cases where they do have care packages, as in those packages respite care is included and this has no financial implications for the elderly or the caregiver.

*Q13: “He is going to a day center, two times a week. (…). During those two days, I can recover a bit, I try to recover from my busy week…” (caregiver Belgium, Brussels)*

In the subunits where respite care was used, it was very much appreciated and it allowed the caregiver to have some time for themselves or their families.

*Q14: “I told the doctor that my husband should go to a day clinic for rehabilitation. They picked him up and he would see somebody else than always me. Then I can do other things and have some relief. I will get a piece of paper and then it will be possible.” (caregiver Germany)*

*Q15: “Dependent person goes to a day care centre twice a week. They do handicraft, crosswords, cooking (like cookies for Christmas). Dependent person stays there from 9 h30 to 17 h00. Caregiver drives dependent person to the centre, there is also a bus but it never was punctual.” (caregiver Luxembourg)*

The case-context influenced the extent to which caregivers used informal support measures. The way information was provided also influenced the use of the support measures and this varied between the different cases. If the caregivers were provided with more information, this resulted in an increased use of formal and informal support measures.

The sources of information varied between the cases. In the Belgian case, the informal caregivers or the dependent elderly really had to search for information themselves.

*Q16: “I didn’t even know that these support measures exist. I should have known about their existence before.” (caregiver Belgium, French speaking).*

*Q17: “It's really looking for things and calling around, getting to know/ finding out. And then you see peers left and right from you who experience the same problems, the same situation and you work it out. You really have to persevere and repeatedly fil out forms.” (caregiver Belgium, Dutch speaking).*

In the cases where information was included within a care package, it was provided through the health insurance, their GP, retirement homes, private nurses, social workers (notably those working in municipal social centres (France), word of mouth communication, or local support group*.*

*Q18: “The nursing help is part of your insurance package. And the cleaning, that depends on your income and you pay a monthly contribution. And the rest is paid by the council (ACTD). You must at least pay a contribution.” (caregiver The Netherlands).*

*Q19: “The “Assurance Dépendance” gave us about €1000 a month because I was taking care of him, that was when (organisation name) came twice a day. Since he is partly paralysed they come 4 times a day, and we still get about €500 each month.” (caregiver Luxembourg).*

This also means that the informal caregiver or the dependent elderly does not need to look for information themselves, as it has been provided to them in a proactive way. This was provided by health and social care professionals or insurances, which is in contrast with the Belgian case, where information was not easily available and not offered uniformly and ad hoc.

All caregivers, and especially those who lack information, stressed the necessity of being proactively informed instead of having to search for it themselves. Belgian subunits mentioned that often the information was provided by the social assistant at discharge from hospital, too late in the care process.

## Discussion

This study explored the experiences with policy measures of informal caregivers in five European countries: Belgium and four other European countries (France, Germany, The Netherlands, and Luxembourg). The lessons learned through the eyes of the informal caregivers provide important insights on (1) the accessibility and the use of different policy measures and (2) how the continuity of care for the dependent elderly at home is coordinated and organised.

### The accessibility and the use of support measures

Primary care organisation was unique in the described cases. The different countries in this study had complex and varied policy measures. Therefore results emphasise the differentness of the environment in which support policies for caregivers are implemented and delivered. The use of support measures depends on whether sufficient and timely-appropriate information is communicated to the end users.

Earlier research shows similar results [7, 14, 15]. The use of policy measures (formal care) differs between European countries, but when allocating this formal care every country focuses on older persons living alone or on the most care-dependent persons [14]. Literature describes that the majority of these elderly persons and their carers do not seem to seek for help for their unmet needs [15]. The complexity of finding information is one of this reasons.

Hence, the results from the case studies are in line with what was already reported in literature: caregivers only look for information on what is available to them very late in the care process, and in extreme situations, informal caregivers often seek for information on support for themselves.

As a solution for the problems described, the use of integrated care packages is recommended. Integrated care packages by health insurance or by the municipality (as for instance in the cases of France and the Netherlands) facilitate transfer of knowledge on informal support measures to the end users which can increase the use. In the Belgian case, fragmentation of information about policy measures is apparent. Caregivers, who are engaged in a complex caring micro-environment, are obliged to search for information themselves. A mixture of information is available to adults with chronic diseases. Because of the different health care professionals connected to these patients, this information is fragmented and the search for information is very difficult [16].

### Organisation and coordination of continuity of care

From our data it shows that most informal caregivers organize the coordination of care themselves to ensure continuity of care for their elderly. Formal support measures help the caregiver to offer support to the elderly person. Informal caregivers often experience difficulties in ensuring the continuity of care. Often this continuity is a fragile equilibrium, due to the high age of the caregiver or the combination of working and caring for their family member.

As we know, risk factors for caregiver burden are frequently described in literature [17, 18]. Caregiver burden can threaten the coordination of care for the elderly person. One of the risk factors is the lack of information and support for the informal caregiver.

Therefore, the provision of information and the support in finding custom-fit information for the caregiver has to improve. Information about policy measures should be available to support the caregiver, in order to keep the dependent elderly at home longer. Our study showed that integrated care packages, where information is tailored, are effective in the support for the caregiver [8]. Not only were the accessibility and the use of the support measures increased, but the caregivers also experienced a lot of support when information was provided early in the care process.

Care packages, where a case manager acts as a central contact person and offers information “just in time”, can be a valuable support for the informal caregiver in specific situations. The GP and/ or other health care professionals (e.g. nurse, social assistant, or pharmacist) can fulfil this role of case manager. The case manager must guide the informal caregivers and their dependent elderly person. They need to maintain an overview of the situation of the caregiver, provide information, and look whether the situation is still bearable and doable for the caregiver.

Wagner et al. identified the role of case management to provide this information [19]. Such case management in the community facilitates optimal chronic care management by enhancing the coordination of home care providers from the health and social care sector.

### Strengths and limitations

Even though a uniform but heterogeneous blueprint for the recruitment of participants in the different cases was designed, problems with recruitment in the different cases did not allow the researchers to completely follow this initial strategy. This was particularly the case in Luxembourg, where only retired caregivers could be recruited.

Since participation was voluntary a selection bias is possible, but it is unclear whether this influences the perception of caregivers to support measures.

The intention was to interview dependent elderly and informal caregivers in pairs (subunits). In more than a half of the cases, caregivers and their dependent elderly person were actually both interviewed. The interview with the dependent elderly was done supplementary, after the interview with the caregiver. These interviews did not bring new insights, but rather recapitulated the data given by the informal caregiver and confirmed the caregivers’ experiences, regarding the use of policy measures and the need for support.

A selected number of individuals were interviewed in the different countries. Their specific experiences cannot reflect the overall picture of informal care in each country; but nevertheless the results allow the researcher to test whether the first explanations emerging from one case are robust enough when applied to other cases. In this way, within the aim of the research, an overall description of the caregivers’ experiences brought valuable insights. Based on these experiences, we can learn about the factors that influence the uptake and impact of the different policies.

## Conclusions

The informal caregiver plays an essential role in the long term care. Support to informal caregivers is a complex issue. Each caregiving situation is unique. Therefore, a single support strategy cannot benefit all informal caregivers and tailored measures are necessary. Measures to support informal caregivers are scattered in different sectors of the social security system. Not surprisingly, developing a coherent policy to support informal caregivers is not a simple task and a forward-looking approach is required.

To support the informal caregiver, who is the best person to monitor the progression of the patient’s problem, fragmentation of information regarding supportive policy measures is an issue of concern. Fragmentation of the system is more prominent in Belgium compared with other countries and Belgian informal caregivers find it difficult to navigate through the system. In the other countries, when home care is provided via a care package, information seems to be given more timely, and/or there is a regular assessment of the changing needs of the dependent elderly.

## Abbreviations

ADL, activities of daily living; DEP, Dependent elderly; GP, General practitioners; OECD, Organisation for economic cooperation and development; UZA-UA, University Hospital of Antwerp, University of Antwerp

## Additional file

Additional file 1: Table S1. Summary of support measures to avoid loss of income, of social security benefits or of employment. This table provides an overview of the existing support measures to avoid loss of income of social security benefits or of employment. (DOCX 14 kb)

## Appendix

Interview script and probing questions of the semi structured interviews with the caregivers and their dependent elderly. This table provides an overview of the interview script and the probing questions of the semi-structured interviews with the caregivers and their dependent elderly.

**Table 3:** Interview script and probing questions of the semi structured interviews with the caregivers and their dependent elderly

Introduction question: “*We are conducting a study on the current situation of the caregiver in different European countries, especially on what supports them in playing their role at different levels (economic, employment..)* “*We are interested in your personal experiences and perceptions because you are probably the best placed to tell us on how you feel the caregiver is supported by different policies in this country and also in this region…*
Part 1 : Description of the family, professional and living situation of the ICG (and of the DEP) Question 1 : “*We’ll begin with some questions relating to you and your family, can you tell me something more about yourself (your age, children,…) can you tell us what is the composition of your family* ?” Question 2 : “*Do you live here, with*… (the dependent elderly she/he cares for) ? Question 3 : “*Could you describe your current job situation or (previous) occupation* (voluntary work) ?” Question 4 : “*Has your situation changed since you’ve been caring (for the elderly) and how* ?”
Part 2 : Caregiver role Question 5 : (care history) “*Since when did you start to take care of … OR “How long have you been providing care for the* dependent elderly?” Question 6 : “*How and why did you have to start caring for the* dependent elderly*? Can you tell me something more about it, what led you to take up this role*?” Question 7 : “*Are there other people you are looking after or take care of? (because of disability because of old age, sickness, mental health problems,…* )?” Question 8 : “*Are/were you the main caregiver of … ? Are there other people who help you with taking care of the elderly ? another family member, friends*,… ?” Question 9 : “*Can you tell me a little bit more about your role as caregiver, how do you feel about it? Prompts: what are the positive experiences of this caregiving role and what are the negative experiences* ?” Question 10 : “*As caregiver, which tasks are you doing specifically* (tasks you are doing *for the dependent elderly because he/she can’t do these anymore…) ?”* Question 11 : “*According to you, how much time do you spend doing these tasks /per week* (in general, not per task) ?” Question 12 : “How do you feel about doing these tasks? (prompts: *are some of these tasks more problematic than others and why? Which tasks do you feel most comfortable with and why*?”) Question 13 : “*How do these care tasks affect your daily life* ?” Question 14 : “*How do you cope with it ? Are there other family members or friends that help you with this ?*
Part 3: Formal services for the dependent elderly Question 15 : “*Which are the (professional) services (the dependent elderly) receive at home ?, Are there other arrangements with domestic help, health professionals, ICT support (alarm system,…), adaptation to the house, other (undeclared worker)* ? Question 16 : “*How has this changed (in term of intensity, frequency…) during the last six months* ?” Question 17 : “*Have you experienced some situations in which these formal services that support you were interrupted or could not be delivered ? For example a nurse who can’t come, an acute situation arise and the dependent elderly you care for needs suddenly more care, or the cleaning who doesn’t come? How do you deal with it*?” Question 18 : “*How are the costs of the professional services covered for* ?” Question 19 : “ How could the services be improved in order to support you as an informal caregiver, (prompts: *Are there some services that should be helpful (from your own point of view) for the person you care for, but he/she doesn’t receive? Why (financial reasons, poor quality, accessibility issues, values, bad reputation, doesn’t want to*)?”
Part 4: Formal services for the informal caregiver Question 20 : “*Do you need some care services at home for yourself* ?” Question 21 : “*Are there some services you use or not use that allow you to take a break from your care task or when you are ill or to allow you to go on holiday? why or why not? How did you find out about these services*?” Question 22 : “*What would be helpful for you as caregiver* ?” Question 23 : “*Are there some support services or information that you have used to help you as a caregiver in looking after the dependent elderly*?” Whom did you receive help from? Why and why not? In how far has this helped you ? What way ? Question 24: “*Do you consider that informal care givers are sufficiently informed concerning possible helps* (financial, material, organisational, administrative....)? “ Question 25 : “*How are you informed? Who provided this information*?” Question 26 : “*How has your role as caregiver affected your financial and or work situation? What could help you and/or what has helped you in what way in your financial and employment situation*?” Question 27 : “*Have you considered receiving financial remuneration ? How and why ? How would this help you* ?” Question 28 : “*In what way can this change you relationship with the elderly*?” Question 29 : “*Do you benefit from other measures that has helped you in your role as caregiver*? Question 30 : “*Are the services that support you at the moment enough to help you to care for the dependent elderly and to keep he/she at home in the long term* ?” Question 31 : “How is your role as informal caregiver recognized?

## References

[1] OECD. Health at a glance: Europe 2011. http://ec.europa.eu/health/reports/docs/health_ glance_en.pdf. Accessed December 8, 2012.

[2] Van Durme TMJ, Anthierens S, Symons L, Schmitz O, Paulus D, den Heede V, Remmen R (2014). Stakeholders' perception on the organization of chronic care: a SWOT analysis to draft avenues for health care reforms. BMC Health Serv Res.

[3] Colombo F, Llena-Nozal A, Mercier J, Tjadens F (2011). Help Wanted? Providing and paying for Long-Term Care OECD.

[4] Criel B, Vanderberghe V, De Koker B, Decraene BEE, Waltens R (2014). Informal home care for elderly in Belgium: A study on the Features and challenges of informal care at local level. Community Ment Health J.

[5] Levine C, Halper D, Peist A, Gould DA (2010). Bridging Troubled Waters: Family caregivers, transitions, and long-term care. Health Aff.

[6] Garcés K, Carretero S, Rodenas F, Aleman C (2010). A review of programs to alleviate the burden of informal caregivers of dependent persons. Arch Gerontol Geriatr.

[7] Anthierens S, Willemse E, Remmen R, Schmitz O, Macq J, Declercq A, Arnaut C, Forest M, Denis A, Vinck I, Defourny N, Farfan-Portet MI (2014). Support for informal caregivers – an exploratory analysis. Health Services Research (HSR).

[8] Lopez-Hartmann M, Wens J, Verhoeven V, Remmen R (2012). The effect of caregiver support interventions for informal caregivers of community-dwelling frail elderly: a systematic review. Int J Integr Care.

[9] Washington KT, Meadows SE, Elliott SG, Koopman RJ (2011). Information needs of informal caregivers of older adults with chronic health conditions. Patient Educ Couns.

[10] Yin RK (2009). Case study research: Design and methods (Fourth edition ed. Vol. 5).

[11] Bleichrodt H, van den Berg B, Eeckhoudt L (2005). The Economic Value of Informal Care: A Study of Informal Caregivers' and Patients' Willingness to Pay and Willingness to Accept for Informal Care. Health Econ.

[12] Hoefman R, van Exel N, Brouwer W. MTA Valuation of Informal Care Questionnaire (iVICQ). 2011; http://www.bmg.eur.nl/fileadmin/ASSETS/bmg/english/iMTA/Publications/Manuals___Questionnaires/iVICQ_UK.pdf. Accessed March 8,2015.

[13] Hoff A, Hamblin K. Carers between Work and Care. Conflict of Chance. Oxford: University of Oxford Institute of Population Ageing; 2011. http://www.carersatwork.tu-dortmund.de/download/National%20report%20PL.pdf. Accessed October 15, 2014.

[14] Geerts J, Van den Bosch K (2012). Transitions in formal and informal care utilisation amongst older Europeans: the impact of national contexts. Eur J Ageing.

[15] Walters C, Iliffe S, Orrell M (2001). An exploration of help-seeking behaviour in older people with unmet needs. Fam Pract.

[16] Manderson B, Mcurray A, Piraino E (2012). Navigation roles support chronically ill older adults through healthcare transitions: a systematic review of the literature. Health Soc Care Community.

[17] Sansoni J, Anderson KH, Varona LM, Varela G (2013). Caregivers of Alzheimer's patients and factors influencing institutionalization of loved ones: some considerations on existing literature. Annale di inguini.

[18] Adelman RD, Tmanove LL, Delgado D, Dion S, Lachs M (2014). Caregiver burden: a clinical review. J Am Med Assoc.

[19] Wagner EH, Austin BT, Davis C, Hindmarsh M, Scheafer J, Bonomi A (2001). Improving chronic illness care: translating evidence into action. Health Aff.

