# Supplementary material for: Do informal caregivers for elderly in the community use support measures? A qualitative study in five European countries
Source: BMC Health Serv Res. 2016 Jul 16;16:270. doi: 10.1186/s12913-016-1487-2 (PMC4947246; doi:10.1186/s12913-016-1487-2)
Supplement: Additional file 1: Table S1. — Summary of support measures to avoid loss of income, of social security benefits or of employment. This table provides an overview of the existing support measures to avoid loss of income of social security benefits or of employment. (DOCX 14 kb) [file 12913_2016_1487_MOESM1_ESM.docx]

**Additional file 1**

**Table: Summary of support measures to avoid loss of income, of social security benefits or of employment**

| **Type of measure** | **Belgium** | **France** | **The Netherlands** | **Germany** | **Luxembourg** |
| --- | --- | --- | --- | --- | --- |
| **Caregivers’ allowance** | *Mantelzorgpremie* (only in Flanders)  Beneficiaries in 2012: 30 242 | No | *Mantelzorgcompliment* Beneficiaries in 2012: 377 313 | No | No |
| **Dependent elderly using the cash-for-care allowance to compensate the caregiver** | *Vlaamse zorg-verzekering*  Beneficiaries in 2006: 20%  *Allocation pour l’aide aux personnes âgées – (APA – THAB)*  Beneficiaries: N.A. | *Allocation personnalisée d'autonomie (APA)*  Beneficiaries 2008: 16% | *Persoonsgebonden budget*  Beneficiaries in 2006: 33% | *Pflegegeld*  Beneficiaries : N.A. | *Prestation en espèces*  Since the beginning of the dependency insurance: 496 people received a labour contract |
| **Pension contributions** | Limited.  When: leave from work or if a labour contract is signed  Beneficiaries: N.A. | Limited.  When: leave from work or if a labour contract is signed  Beneficiaries: N.A. | *Mantelzorgforfait*  When: paid to informal caregivers if they are compensated via the *Persoonsgebonden budget*  Beneficiaries: N.A. | Paid to informal caregivers (subjected to certain eligibility) Beneficiaries, 2012: 414 000 actively insured | Paid to informal caregivers (subjected to certain eligibility) Beneficiaries, since the establishment of the measure: 2545 |
| **Unemployment benefits for caregivers** | Extension of rights: No duty to look for employment, refuse a job offer and no need to be inscribed as looking for employment.  Beneficiaries: N.A. | No  Beneficiaries: N.A. | *Mantelzorgforfait* paid to informal caregivers if they are compensated via the *Persoonsgebonden budget*  Extension of rights: No duty to look for employment  Beneficiaries: N.A. | Unemployment benefits are granted when in unpaid leave.  Unemployment contributions can be paid on a voluntary basis by the informal caregiver.  Beneficiaries: N.A. | No |
| **Specific paid work leave** | Yes  Beneficiaries in 2012: 11 443 | No | No | No | No |
| **Specific unpaid work leave** | Available via collective agreements  Beneficiaries: N.A. | *Congé de soutien familial*  Beneficiaries: N.A. | Available via collective agreements  Beneficiaries: N.A. | *Plegezeitgesetz*  Beneficiaries: N.A. | Available via collective agreements  Beneficiaries: N.A. |
| **Flexible work arrangements** | Yes, through use of part-time leave  Beneficiaries: N.A. | No, but available via collective agreements  Beneficiaries: N.A. | No, but available via collective agreements  Beneficiaries: N.A. | *Familienpflegezeit*  Beneficiaries: N.A. | No, but available via collective agreements  Beneficiaries: N.A. |
